# Heat exposure promotes sarcopenia via gut microbiota‐derived metabolites

**DOI:** 10.1111/acel.14370

**Published:** 2024-10-29

**Authors:** Yi‐Fan Guo, Zhe‐Yu Liu, Min Zhou, Wei‐Hong Kuang, Ya Liu, Yan Huang, Ping Yin, Zhu‐Ying Xia

**Affiliations:** ^1^ Department of Endocrinology, Endocrinology Research Center Xiangya Hospital of Central South University Changsha Hunan China; ^2^ Department of Oral and Maxillofacial Surgery, Center of Stomatology,Xiangya Hospital Central South University Changsha Hunan China; ^3^ National Clinical Research Center for Geriatric Disorders Xiangya Hospital of Central South University Changsha Hunan China

**Keywords:** gut microbiota, homocitrulline, sarcopenia

## Abstract

The unprecedented rise in global ambient temperatures in the last decade has significantly impacted human health, yet how heat exposure affects the development of sarcopenia remains enigmatic. Here, we demonstrate that chronic heat exposure induces skeletal muscle volume loss, leading to muscle strength and functional decline in mice. The microbiota composition of heat‐exposed mice was analyzed using 16S ribosomal DNA analysis. Liquid chromatography‐mass spectrometry (LC–MS) was used to explore the effects of heat exposure on the blood metabolome and to further analyze the correlation between blood metabolism and gut microbiota. Transplantation of microbiota from heat‐exposed mice to germ‐free mice was sufficient to increase adverse effects on skeletal muscle function in the host. Mechanistically, using an untargeted metabolomics strategy, we reveal that altered gut microbiota due to high temperatures is associated with elevated serum levels of homocitrulline. Homocitrulline causes mitochondrial dysfunction in myocytes by exacerbating ferroptosis levels. And Nrf2 activator (Oltipraz) supplementation alleviates muscle atrophy and dysfunction induced by heat exposure. Our findings reveal the detrimental effects of heat exposure on muscle function and provide new strategies for treating sarcopenia.

## Graphical abstract

Heat exposure leads to significant changes in the composition of the gut microbiota. Mechanistically, heat‐induced changes in the gut microbiota are associated with elevated serum levels of Hcit. Hcit causes mitochondrial dysfunction in myocytes by exacerbating ferroptosis levels. Nrf2 activator (oliprazole) reversed this process.

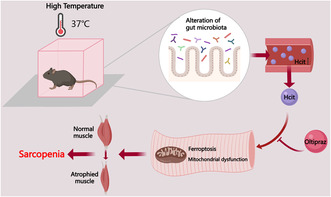

**Abbreviations**
AREs: antioxidant response elements
CLAMS: Comprehensive Laboratory Animal Monitoring System
CSA: cross‐sectional area
FMT: fecal microbiota transplantation
FR: food restriction
GF: germ‐free
GSH: glutathione
HT: high temperature
IS: indolyl sulfate
LC‐MS: Liquid chromatography‐mass spectrometry
LEfSe: Linear discriminant analysis effect size
MDA: Malondialdehyde
MMP: mitochondrial membrane potential
NMDS: non‐metric multidimensional scaling
PCA: Principal component analysis
ROS: reactive oxygen species
RT: room temperature
SCFAs: short‐chain fatty acids
SDH: Succinate dehydrogenase
UHI: urban heat island
ΔψM: Mitochondrial membrane potential

## 1 INTRODUCTION

In response to the present climate change, more and more studies are focusing on the relationship between changes in the climatic environment and human health, and some research has been done on the link between high‐temperature exposure and metabolism. High temperature is a causative factor for multi‐system diseases such as respiratory, circulatory, urinary, and neurological systems (Anderson & Bell, 2009). The occupational population in high‐temperature environments is more vulnerable to health damage due to production mode, labor intensity, and workplace heat source (Foster et al., 2020). Sarcopenia is a progressive skeletal muscle disease characterized by a reduction in muscle mass and strength (Cruz‐Jentoft et al., 2019). Studies confirm that heat exposure affects muscle health; for example, heat exposure reduced upper body endurance work capacity and maximal arm and leg strength in young men (Otani, 2023). In addition, acute heat stress causes oxidative stress damage in muscles (Chang et al., 2022), but the specific mechanistic link between heat exposure and sarcopenia is unknown.

When faced with a hyperthermic environment, organisms tend to adopt a series of defence‐regulating measures, in which the microbiota plays a vital role (Ubeda & Pamer, 2012). A favorable microbiota can help maintain the gut barrier and effectively ensure the host's health. In contrast, an imbalanced microbiota promotes disease development (Guarner & Malagelada, 2003). High‐temperature environments have been found to induce dysbiosis of the gut microbiota, increased bacterial translocation, mild enteritis, and elevated systemic levels of the pro‐inflammatory cytokine TNF‐α (Chen, Zheng, et al., 2019; Wen et al., 2021). Evidence supports the association of the gut microbiota with the development of sarcopenia (Wu et al., 2020). For example, depletion of gut microbiota directly induced muscle wasting, as evidenced by increased fatigue and reduced muscle strength in germ‐free (GF) mice (Lahiri et al., 2019), while *Lactobacillus casei LC122*, *Lactobacillus paracasei PS23*, and *Bifidobacterium longum BL986* supplements restored muscle loss in aged rodents (Chen, Huang, et al., 2019; Ni et al., 2019). Microbial‐related metabolites also have different effects on skeletal muscle. For example, indolyl sulfate (IS) and LPS accelerated muscle atrophy (Sato et al., 2016; Song et al., 2020), and short‐chain fatty acids (SCFAs) improved mitochondrial activity (Lahiri et al., 2019). However, whether and how heat exposure affects the microbiota still requires further research.

Sarcopenia has a complex multifactorial pathogenesis involving not only age‐related changes in neuromuscular function (Deschenes et al., 2010), muscle protein turnover, hormone levels, and sensitivity (Morley & Malmstrom, 2013), but also chronic pro‐inflammatory states (Bano et al., 2017), oxidative stress (Nemes et al., 2018), and behavioral factors, particularly nutritional status, and physical activity level. Mitochondrial dysfunction in skeletal muscle cells is recognized as a significant driver of sarcopenia (Ferri et al., 2020). Oxidative stress and reduced antioxidant defenses in mitochondria form a vicious cycle that leads to mitochondrial dysfunction.

In this study, we verified the occurrence of sarcopenia induced by high temperature exposure at 37°C in a mouse model, and demonstrated the involvement of intestinal microbiota and related metabolites in this process through intestinal microbiota transplantation. Mechanistically, we used 16 S rDNA in combination with untargeted metabolomics to focus on a metabolic derivative of gut microbiota, homocitrulline. Elevated homocitrulline mediates the occurrence of skeletal muscle ferroptosis via the Nrf2‐Gpx4 axis, along with high intracellular ROS levels and mitochondrial dysfunction. The addition of NRF2 activator Oltipraz reversed the above phenotypes and ameliorate saropenia. These results suggest that high‐temperature exposure accelerates the development of sarcopenia, in which intestinal microbial derivatives play a crucial role.

## 2 RESULTS

### 2.1 Heat exposure promotes sarcopenia in mice

**FIGURE 1:**
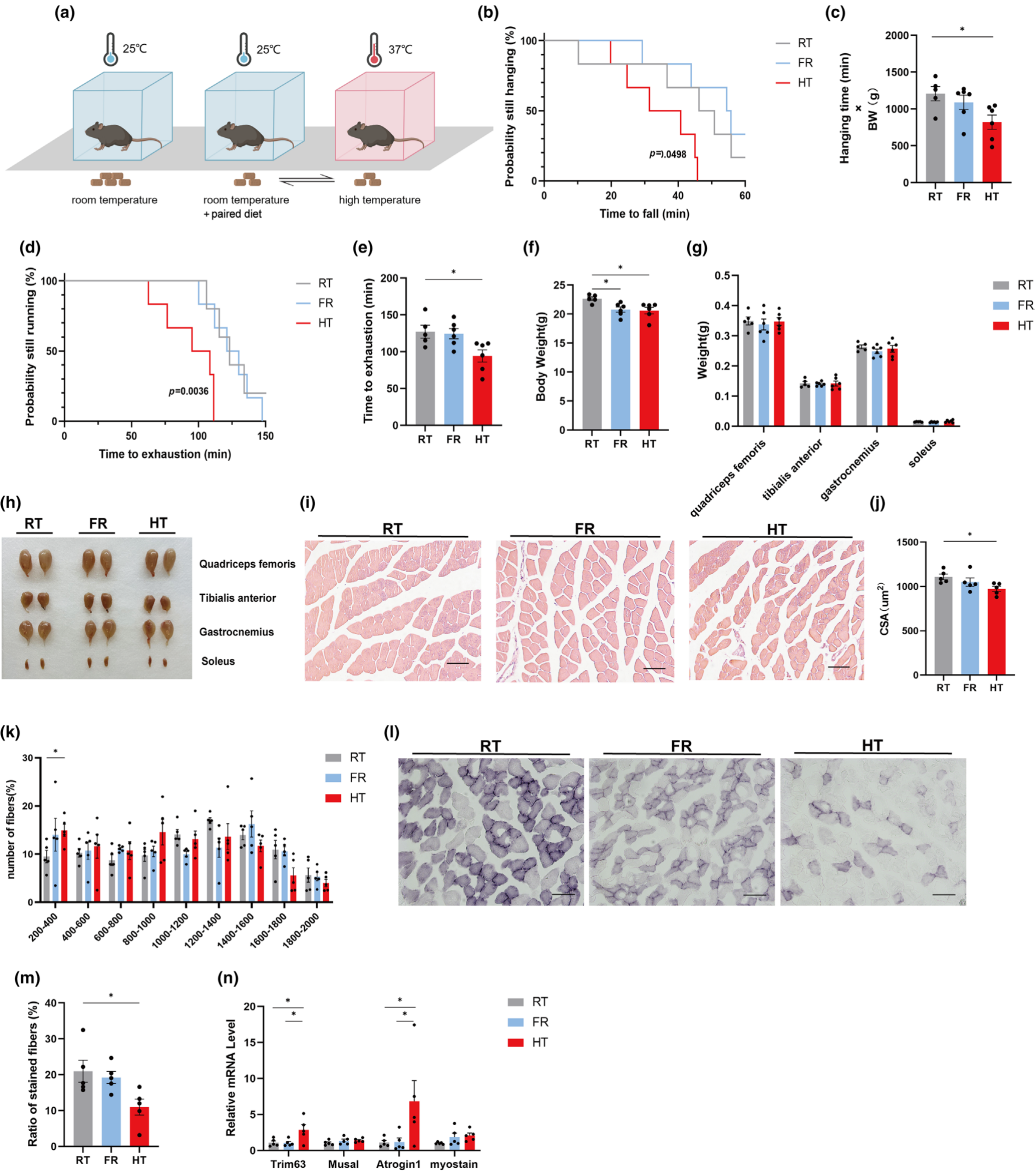
Exposure to high temperature aggravates sarcopenia in mice. (a) Experimental scheme. 12‐month‐old mice were exposed to room temperature (RT), high temperature (HT) and room temperature + paired diet with HT group for 14 days (*n* = 5 or 6). (b, c) Determination of exercise capacity by grip test (*n* = 5–6); (d, e) Determination of exercise capacity by treadmill test (*n* = 5–6); (f) Body weight of three groups mice (*n* = 5–6); (g, h) Weight and representative pictures of dissected quadriceps femoris, tibialis anterior, gastrocnemius and soleus muscles (*n* = 5–6); (i–k) Representative images of HE staining of gastrocnemius muscle cross‐section (i), quantification of mean cross‐sectional areas (j) and distribution of muscle fibers (k). Bar = 80 μm. (*n* = 5); (l, m) Representative images of succinate dehydrogenase (SDH) staining of gastrocnemius muscle cross‐section (l) and quantification (m) of stained muscle fibers. Bar = 80 μm.(*n* = 5); (n) Expression levels of muscle atrophy‐related genes by qPCR. (*n* = 5). Data presented are means±s.e.m. *P<0.05. ANOVA with Fisher’s LSD for multiple comparisons.

To explore the relationship between heat exposure and the prevalence of sarcopenia, we validated it in a mouse model. 12‐month‐old mice were exposed to 25°C (room temperature, RT) and 37°C (high temperature, HT). To eliminate the effects caused by the high‐temperature environment on the mice's ingestion levels, we also set up a group with food restriction (FR) exposed to 25°C (Figure 1a). High‐temperature exposure for up to 14 days resulted in HT mice spending less time on the grid (Figure 1b,c), indicating reduced muscle strength, and less time on the treadmill for maximal running (Figure 1d,e), indicating reduced muscular endurance. It is evident that heat exposure leads to reduced strength and function of skeletal muscle in mice.

We further hypothesised that heat exposure reduces muscle tissue mass in mice. After 14 days of heat exposure, the muscle weights of mice in the FR and HT groups did not decrease significantly within a short period despite the decrease in body weight (Figure 1f–h). Interestingly, histological analysis showed a reduction in the gastrocnemius muscle fiber cross‐sectional area (CSA) in HT mice, along with a significant increase in small fibers (200–400 μm^2^) in HT mice, suggesting that the proportion of small fibers in the muscles of hyperthermic mice is increasing (Figure 1i–k). Succinate dehydrogenase (SDH) staining showed reduced oxidative fibers in HT mice (Figure 1l,m). Expression of crucial muscle atrophy‐related genes (such as *Trim 63* and *Atrogin 1*) was increased in the gastrocnemius muscle of HT mice, whereas the change was not significant in FR mice (Figure 1n).

Considering that ambient temperature has a profound effect on body condition, including metabolism and physical activity, we performed indirect calorimetry using the Comprehensive Laboratory Animal Monitoring System (CLAMS). Analyses showed that oxygen consumption, energy expenditure and food intake decreased in mice after heat exposure, but there were no significant differences (Figure S1a–c). We further examined the hormone levels, including epinephrine, cortisol and vasopressin in the mice after 14 days and found no significant differences between these groups, suggesting that the mice did not show signs of stress after high‐temperature stimulation (Figure S1d–f). These results suggest that heat exposure promotes muscle atrophy and inhibits muscle function and is ingestion‐independent.

### 2.2 Heat exposure alters the composition of the gut microbiota

**FIGURE 2:**
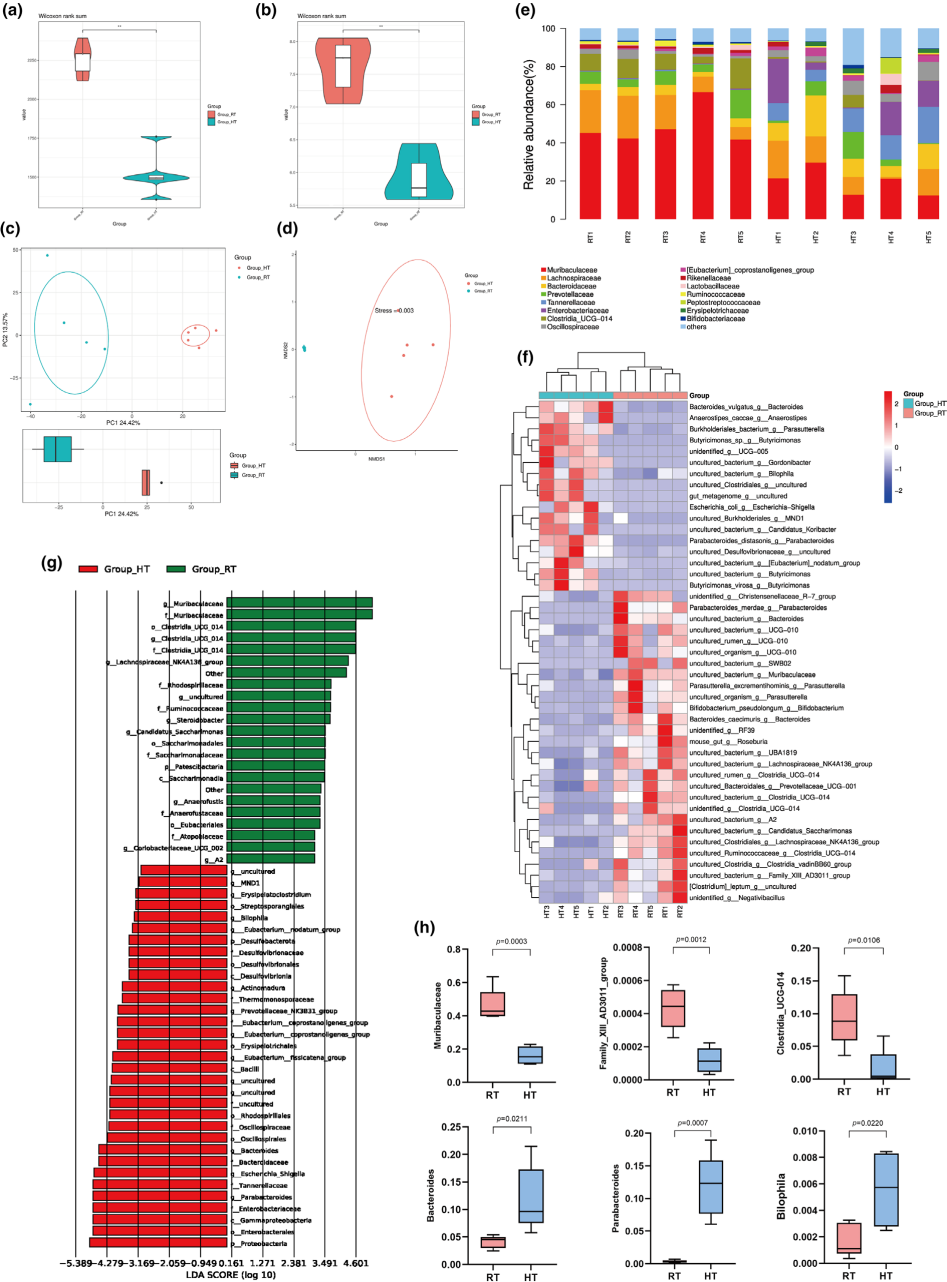
Heat exposure alters microbiota composition. (a, b) Shannon diversity and Chao1 of 16S rDNA sequencing on fecal microbiota of RT and HT mice; (c, d) PCA and NMDS based on 16S rDNA sequencing; (e) Bar chart of the relative microbiome abundance at family level; (f) Hierarchically‐clustered heatmap of differentiated distributed microbiota (*p*<0.05) at species level; (g) Microbiota with top 10 LDA score in each group based on LEfSe analysis. Log10 (LDA score) were shown; (h) Decreased and increased genera in HT group. Data presented are means±s.e.m. **P<0.01. Unpaired t test for binary comparison.

Studies have shown that ambient temperature significantly affects the gut microbiota (Chevalier et al., 2015, 2020; Ziętak et al., 2016). Given that the expression of muscle atrophy‐related genes in FR mice did not differ from RT, we collected faeces from RT and HT mice to study the effects of heat exposure. The microbiota composition was analysed by 16S ribosomal DNA analysis. Based on the results of alpha‐diversity (including Chao1 and and Shannon indices, which indicate the abundance and homogeneity of the community), the HT group was shown to be low in both abundance and homogeneity (Figure 2a,b), indicating a less even distribution in abundance of the bacterial species after heat exposure. Principal component analysis (PCA) and non‐metric multidimensional scaling (NMDS), representing β‐diversity, showed significant changes in the microbiota content of faecal samples in the HT group (Figure 2c,d).

Previous study revealed that the predominance of the *Muribaculaceae* family was dampened in the warm‐adapted microbiota (Chevalier et al., 2020). The reduced specific abundance of *Muribaculaceae* in the HT group further validated the sensitivity of this microbiota to high temperature (Figure 2e). Hierarchical clustering heat maps representing differentially distributed microbiota at the species level also showed that heat exposure resulted in significant alterations in the gut microbiota of mice (Figure 2f). In addition, we performed shotgun macrogenomic sequencing of these fecal materials. Linear discriminant analysis effect size (LEfSe) analyses revealed characteristic bacteria for each group (Figure 2g). It was further verified that the relatively high abundance of object clocks in the HT group was significantly different from the RT group. Among gut microorganisms in HT, *Muribaculaceae*, *Family_XIII_AD3011_group* and *Clostridia_UCG‐014* levels were reduced, *Bacteroides*, *Parabacteroides* and *Bilophila* levels were increased (Figure 2h). Study shows significant reduction in relative abundance of *Muribaculaceae*, *Clostridia_UCG‐014* in patients with sarcopenia (Zhou et al., 2022). *Bacteroides* are involved in a number of important metabolic activities, including the fermentation of carbohydrates, the utilisation of nitrogen‐containing substances and the biotransformation of steroids, and that there is a positive correlation between *Bacteroides* and skeletal muscle mass (Wang et al., 2023). *Parabacteroides* has also been shown to be a bacterial genus that is increased in patients with sarcopenia and can be used as a biomarker for the diagnosis of sarcopenia (Song et al., 2024). Accordingly, heat exposure leads to robust and consistent changes in the gut microbiota composition.

### 2.3 Transplantation heat‐exposed microbiota inhibits muscle function

**FIGURE 3:**
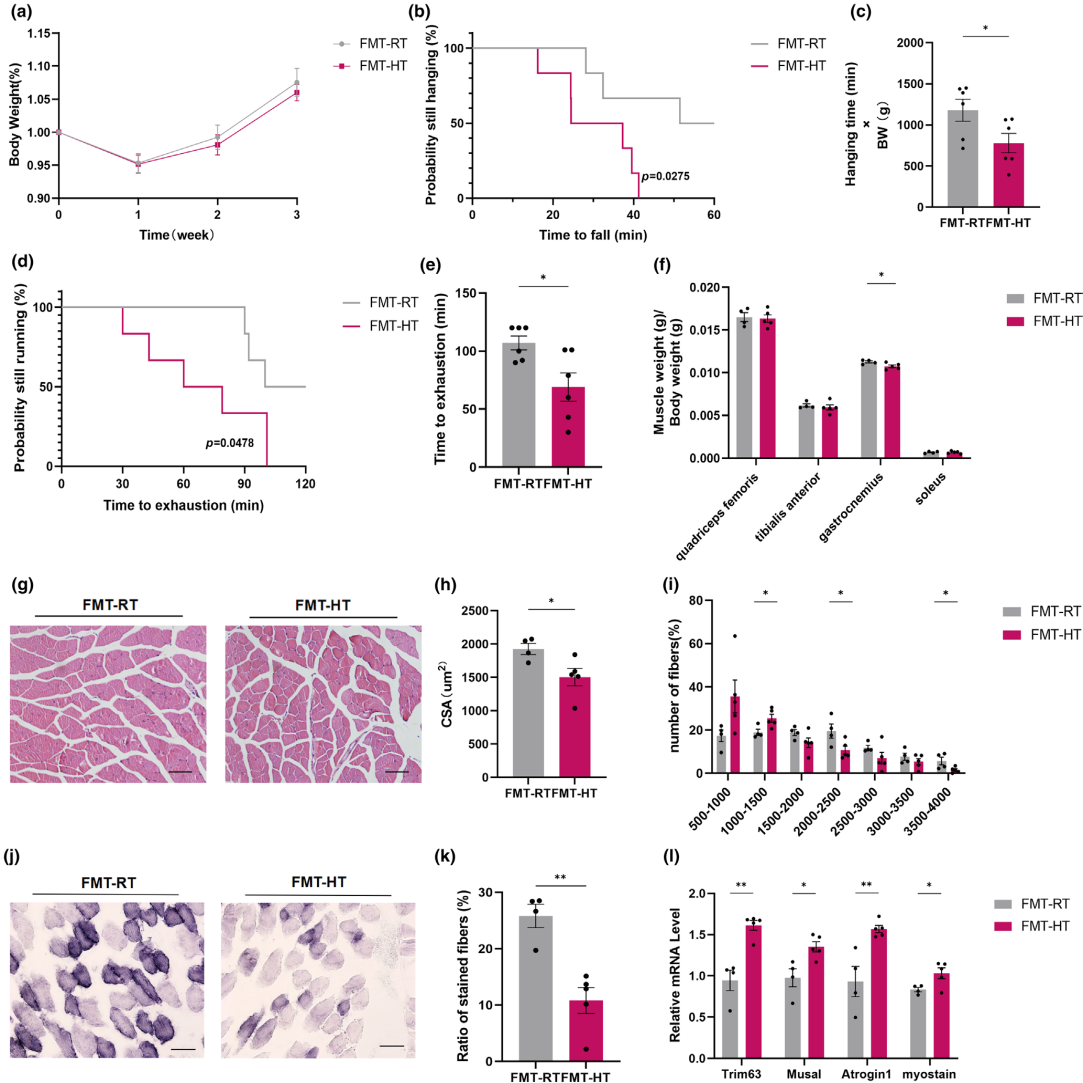
Microbiota transplantation from heat‐exposed treated mice inhibits muscle function. (a) Body weight of FMT‐RT and FMT‐HT mice (*n* = 6); (b, c) Determination of exercise capacity by grip test (*n* = 6); (d, e) Determination of exercise capacity by treadmill test (*n* = 6); (f) Weight of dissected quadriceps femoris, tibialis anterior, gastrocnemius and soleus muscles (*n* = 4–5); (g–i) Representative images of HE staining of gastrocnemius muscle cross‐section (g), quantification of mean cross‐sectional areas (h) and distribution of muscle fibers (i). Bar = 80 μm. (*n* = 4–5); (j, k) Representative images of succinate dehydrogenase (SDH) staining of gastrocnemius muscle cross‐section (j) and quantification (k) of stained muscle fibers. Bar = 80 μm. (*n* = 4–5); (l) Expression levels of muscle atrophy‐related genes by qPCR. (*n* = 4–5). Data presented are means±s.e.m. *P<0.05, **P<0.01. Unpaired t test for binary comparison.

To investigate the importance of microbiota changes during heat exposure, we transplanted the microbiota of HT or control RT mice into germ‐free (GF) mice by fecal microbiota transplantation (FMT) experiment. 12‐month‐old mice were divided into two groups treated with faecal material from RT (FMT‐RT) and HT (FMT‐HT) mice. After 3 weeks, there was a tendency for FMT‐HT mice to lose weight compared to FMT‐RT mice, but there was no significant difference (Figure 3a). Reduced grip capacity and less time on the treadmill for maximal running in the FMT‐HT group compared to the FMT‐RT group (Figure 3b–e). Reduced gastrocnemius muscle mass was also observed in FMT‐HT mice (Figure 3f). Histological analysis showed a significant reduction in CSA, accompanied by an increase in the proportion of small fibers (1000–1500 μm^2^) as well as a decrease in the proportion of large fibers (3500–4000 μm^2^) in the muscles of the FMT‐HT group compared to the control group (Figure 3g–i). At the same time, SDH staining showed a decrease in oxidized fiber in HT mice (Figure 3j,k). Further RT‐qPCR analyses of markers of muscle atrophy revealed that *Trim63*, *Musal*, *Atrogin* and *Myostain* expression was upregulated in FMT‐HT muscles (Figure 3l).

The above results suggest that alterations in the intestinal microbiota induced by heat exposure can lead to muscle atrophy and dysfunction.

### 2.4 Heat exposure causes muscle mitochondrial dysfunction

**FIGURE 4:**
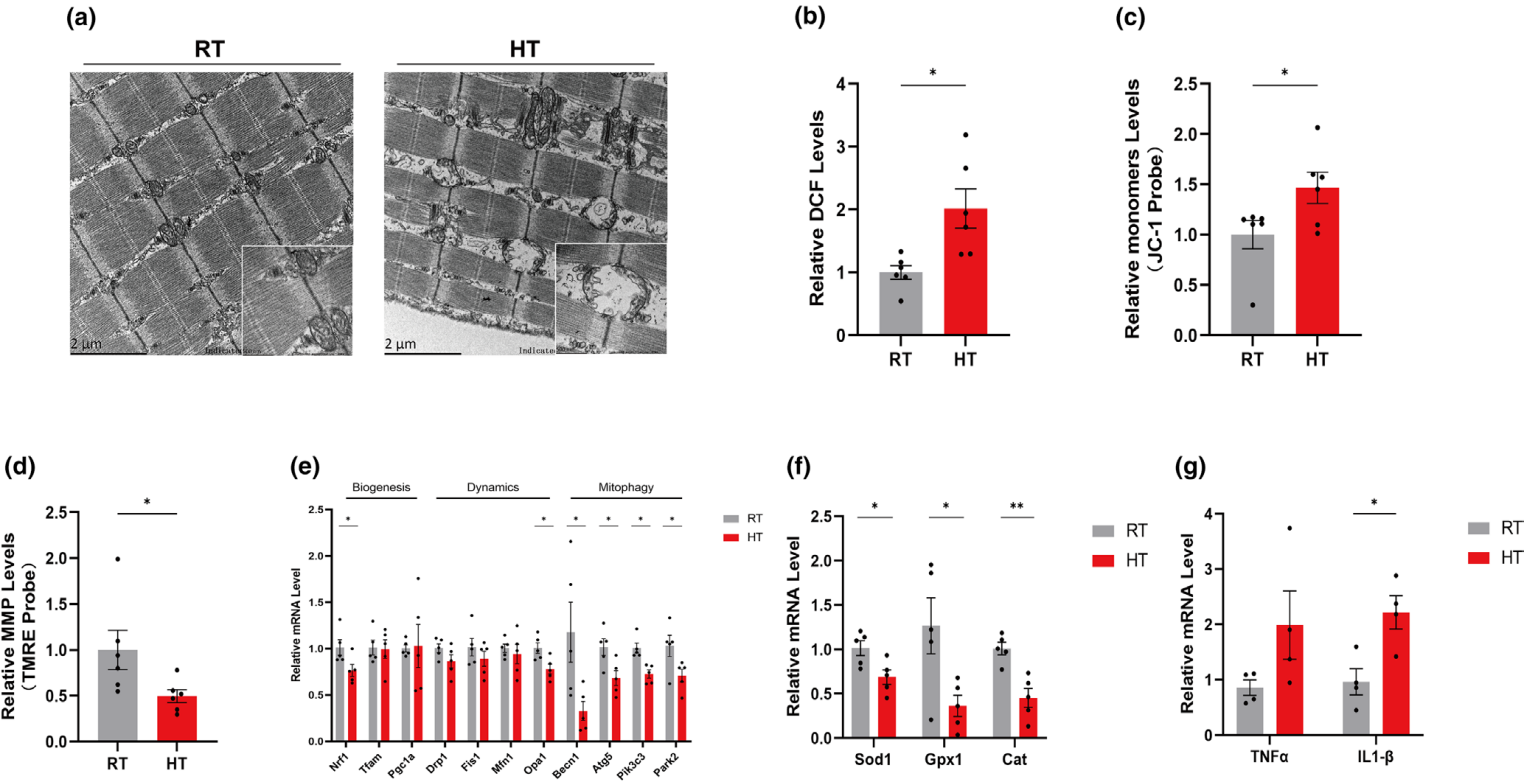
Treatment with high temperatures can lead to muscle mitochondrial dysfunction. (a) Representative transmission electron micrographs (TEM) of intermyofibrillar (IMF) mitochondria from gastrocnemius of RT and HT mice; (b) Determination of ROS level in digested muscle cells through DCF probe. (c, d) Determination of mitochondrial membrane potential (MMP) by JC‐1 probe and TMRE probe in isolated mitochondria from muscle tissue (*n* = 5); (e) Expression levels of mitochondrial biogenesis‐, dynamics‐, and mitophagy related genes by qPCR (*n* = 5); (f, g) Expression levels ofoxidative damage response and inflammation related genes by qPCR (*n* = 5). Data presented are means±s.e.m. *P<0.05, **P<0.01. Unpaired t test forbinary comparison.

Efficient skeletal muscle bioenergetics relies heavily on the metabolic flexibility of mitochondria and strong mitochondrial stimulation, thus effectively integrating mitochondrial function with the rest of the cell and the organism (Cheng et al., 2023; Romanello & Sandri, 2015). Decreased mitochondrial function in skeletal muscle in patients with sarcopenia, accompanied by reduced exercise and physical activity (Johnson et al., 2013). Here, the morphology of mitochondria in mitochondria in the gastrocnemius muscle of HT group mice was found to be significantly changed from compact to rounded and disorganised by electron microscope scanning.(Figure 4a).

The effects of heat exposure on mitochondrial function were then examined. Mitochondria are the main site of reactive oxygen species (ROS) production, and elevated ROS production is closely associated with mitochondrial oxidative damage (Giorgi et al., 2018). With the DCFH probe, we found that HT intervention resulted in elevated ROS levels in gastrocnemius muscle (Figure 4b). We further examined the mitochondrial membrane potential (ΔΨ m), which can be used to determine cellular integrity and function and is a parameter commonly used to determine mitochondrial function (Joshi & Bakowska, 2011). Using the JC‐1 probe and the TMRE probe, increased levels of JC‐1 monomer and decreased levels of TMRE were found in the gastrocnemius muscle of the HT group, suggesting that heat exposure led to a decrease in the level of muscle ΔΨ m (Figure 4c,d). To further investigate the factors influencing mitochondrial dysfunction, we analysed the relevant genes using RT‐qPCR. Genes associated with autophagy and mitophagy, including *Beclin1*, *Atg5*, *Pik3c3*, and *Park2*, were reduced in the heat‐exposed group, accompanied by a decrease in the expression of a number of genes associated with mitochondrial biogenesis (*Nrf1*) and dynamics (*Opa1*) (Figure 4e). Meanwhile, genes related to oxidative damage response (*SOD1*, *GPX1*, *CAT*) decreased and inflammation (*IL‐1β*) increased in HT mice (Figure 4f,g).

These findings suggest that heat exposure impairs mitochondrial function in muscle and also inhibits autophagy and mitophagy as well as antioxidant capacity.

### 2.5 Heat‐exposed mouse gut microbiota‐mediated homocitrulline dysregulation affects muscle mitochondrial function

**FIGURE 5:**
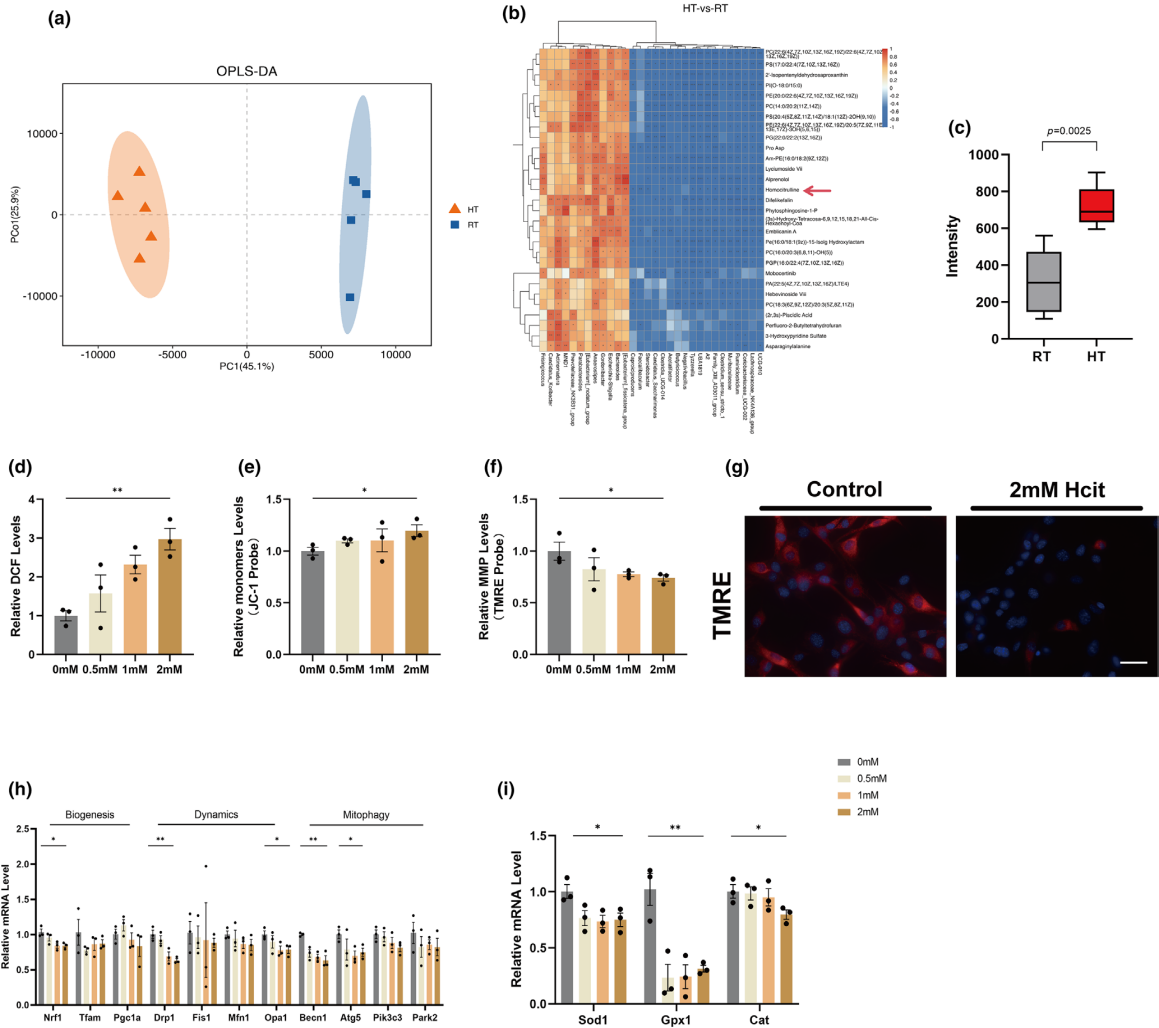
Homocitrulline mediates mitochondrial dysfunction in myocytes. (a) OPLS‐DA of serum metabolite composition in the RT and HT groups; (b) Correlation of intestinal microbiota at species level and the top 30 most important differential biomarkers; (c) Serum Hict levels; (d–g) Determination of ROS, MMP and TMRE in Hcit‐treated C2C12 (*n* = 3). Bar = 20 μm; (h) Expression levels of mitochondrial biogenesis‐, dynamics‐, and mitophagy related genes by qPCR (*n* = 3); (i) Expression levels of oxidative damage response related genes by qPCR (*n* = 3). Data presented are means±s.e.m. *P<0.05, **P<0.01. Unpaired t test for binary comparison. ANOVA with Fisher’s LSD for multiple comparisons.

Gut microbiota plays a crucial role in the digestion and absorption of nutrients and affects blood metabolism (Org et al., 2017). To explore the effects of heat exposure on the blood metabolome and to investigate the correlation between blood metabolism and gut microbiota, we performed metabolomics on serum from RT and HT mice using liquid chromatography mass spectrometry (LC–MS). Orthogonal partial least squares discriminant analysis clearly showed the differences in the metabolites screened (Figure 5a). We then selected the entries in the top 30 metabolites (Table S1) and top 50 microorganisms with the largest significant differences (log_2_(FoldChange)), calculated the correlations between them, and plotted the heatmaps (Figure 5b). Homocitrulline (Hcit) associated with a variety of differential microbiota has attracted our attention. It has been shown that serum levels of Hcit are higher in patients with severe sarcopenia than in non‐sarcopenic subjects (Shin et al., 2022), and that changes in the balance of intestinal flora can regulate serum levels of Hcit (He et al., 2024). Our results also showed an increase in Hcit in heated mice (Figure 5c), which ranked fourth based on the level of differential expression (Supplementary Table S1). We validated this for metabolite that ranked ahead of hypercitrulline and found that Difelikefalin did not alter markers of muscle atrophy in muscle cells (Figure S1g).

To verify whether Hcit causes mitochondrial dysfunction in myoblasts, we intervened C2C12 cells with Hcit at concentrations of 0, 0.5, 1, and 2 mM, respectively. As we expected, the DCFH probe showed that Hcit led to an increase in cellular ROS levels, and the JC‐1 probe and TMRE showed that Hcit led to a decrease in ΔΨ m, and that these changes were strongly correlated with Hcit concentration (Figure 5d–g). Further alterations in genes related to mitochondrial function were detected by RT‐qPCR, and Hcit led to a decrease in the expression of genes related to mitochondrial biogenesis (Nrf1), dynamics (Drp1 and Opa1), autophagy and mitophagy (Beclin1 and Atg5) and oxidative damage response (SOD1, GPX1, CAT) in the cells as the concentration increased (Figure 5h,i). These observations suggest that Hcit causes mitochondrial dysfunction in myocytes and that its effects are positively correlated with concentration.

**FIGURE 6:**
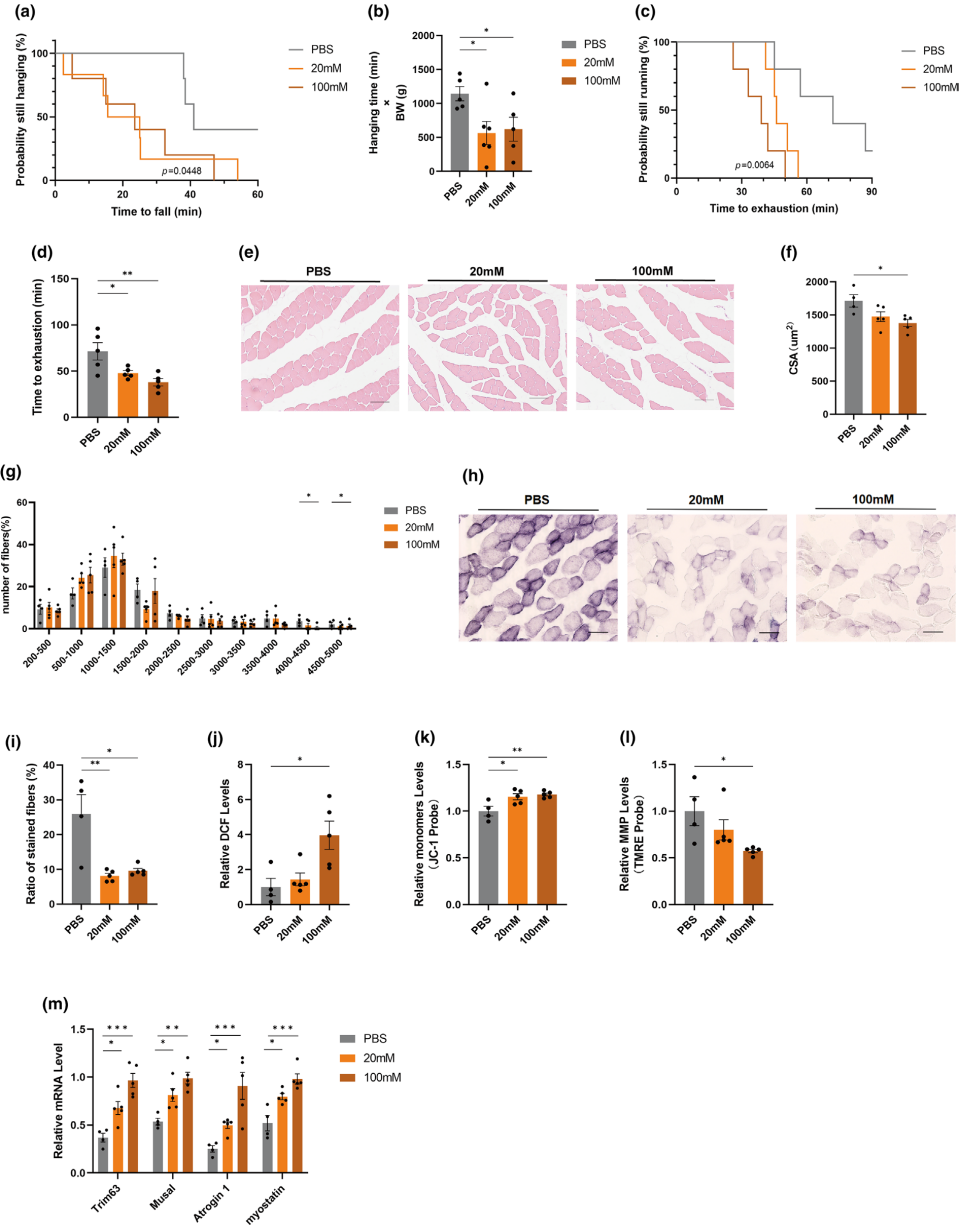
Homocitrulline promotes sarcopenia in mice. (a, b) Determination of exercise capacity by conducting grip test in PBS‐, 20 mM‐ and 100 mM‐treated mice (*n* = 5–6); (c, d) Determination of exercise capacity by conducting treadmill test in PBS‐, 20 mM‐ and 100 mM‐treated mice (*n* = 5–6); (e–g) Representative images of HE staining of gastrocnemius muscle cross‐section (e), quantification of mean cross‐sectional areas (f) and distribution of muscle fibers (g). Bar = 80 μm (*n* = 4–5); (h, i) Representative images of succinate dehydrogenase (SDH) staining of gastrocnemius muscle cross‐section (h) and quantification (i) of stained muscle fibers. Bar = 80 μm (*n* = 4–5); (j–l) Determination of ROS, MMP and TMRE in PBS‐ 20 mM‐ and 100 mM‐treated mice (*n* = 4–5); (m) Expression levels of muscle atrophy‐related genes by qPCR. (*n* = 4–5). Data presented are means±s.e.m. *P<0.05, **P<0.01, ***P<0.001. ANOVA withFisher’s LSD for multiple comparisons.

We further validated the effect of Hcit on muscle function in mice. Every 2 days, 12‐month‐old mice were injected intraperitoneally with 20 mM and 100 mM Hcit, respectively, while another group received PBS as a control. After 3 weeks of intervention, behavioural experimental tests were performed on the mice in each group to assess changes in muscle function and strength. The results showed that Hcit treatment led to a reduction in both the grid suspension time and the maximum running time of the mice (Figure 6a–d). Histological analysis showed that Hcit mice had a reduced CSA of gastrocnemius muscle fibers and a reduced proportion of large fibers (4000–5000 μm^2^) (Figure 6e–g). SDH staining showed reduced oxidised fibers in Hcit‐treated mice (Figure 6h,i). In addition, we detected altered levels of mitochondrial function in the gastrocnemius muscle. Consistent with the cellular results, Hcit‐treated mice showed elevated ROS levels in muscle tissues, while ΔΨ m decreased (Figure 6j–l). The markers of muscle atrophy (*Trim63*, *Musal*, *Atrogin* and *Myostain*) expression was upregulated in Hcit‐treated mice muscles (Figure 6m). These results suggest that heat‐exposed mice with gut microbe‐associated Hcit dysregulation play an important role in muscle mitochondrial dysfunction.

### 2.6 Hcit mediates mitochondrial dysfunction through ferroptosis

**FIGURE 7:**
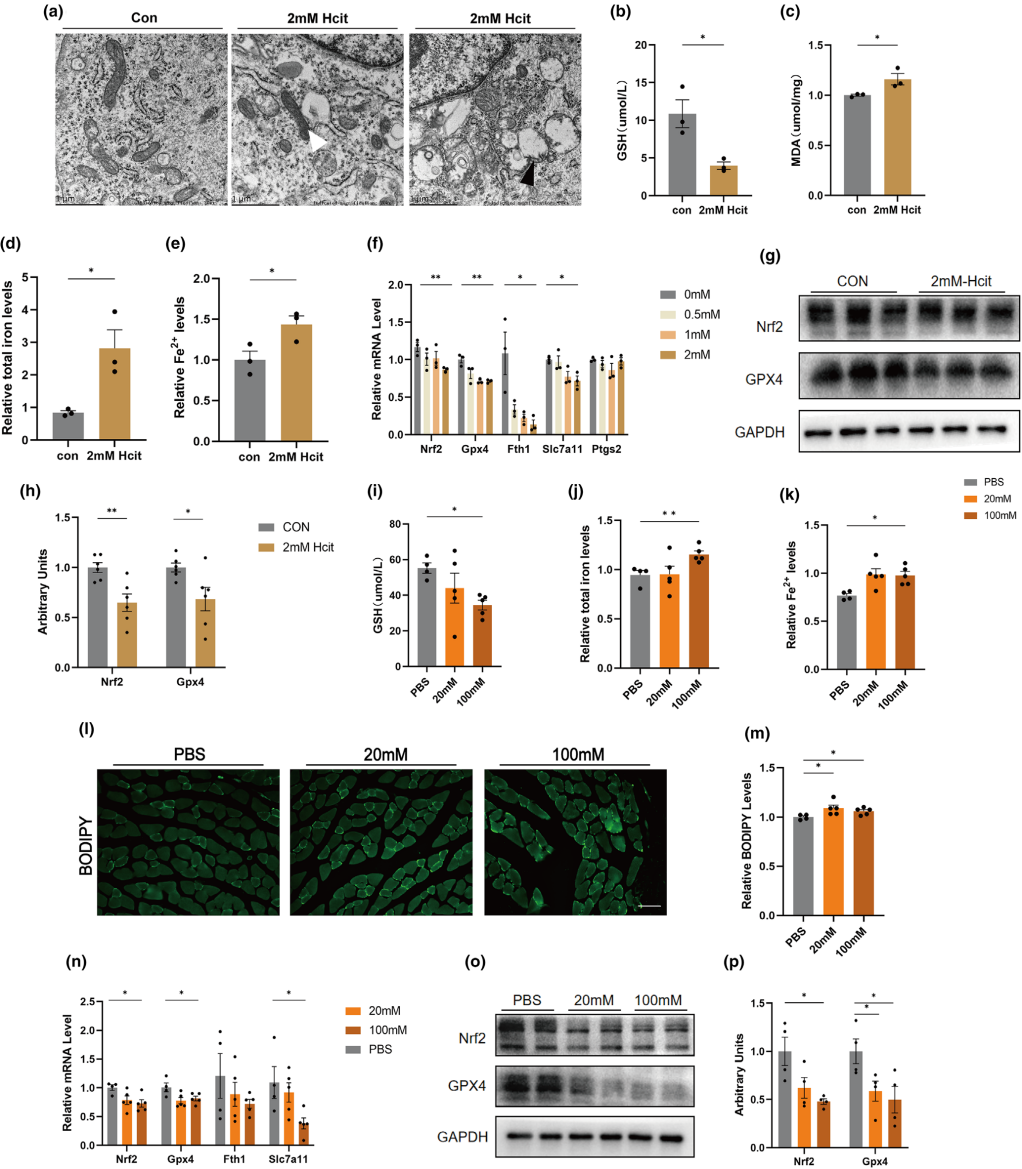
Homocitrulline impairs mitochondrial function by promoting ferroptosis. (a) Representative electron micrographs of Hcit interfering with C2C12. White arrows indicate an increase in the density of the mitochondrial bilayer membrane; black arrows indicate rupture of the outer mitochondrial membrane and disappearance of the mitochondrial cristae; (b, c) Relative GSH and MDA content in PBS‐ and Hcit‐treated C2C12 (*n* = 3); (d, e) Relative total iron and Fe^2+^ levels in PBS‐ and Hcit‐treated C2C12 (*n* = 3); (f) Expression levels of ferroptosis related genes by qPCR (*n* = 3); (g, h) Western blot of ferroptosis related proteins in C2C12 (*n* = 3); (i) Relative GSH content in PBS‐, 20 mM‐ and 100 mM‐treated mice (*n* = 4–5); (j, k) Relative total iron and Fe^2+^ levels in PBS‐, 20 mM‐ and 100 mM‐treated mice (*n* = 4–5); (l, m) Representative images of BODIPY staining of cross section of gastrocnemius muscle (l) and quantification (m) of stained area (*n* = 4–5). Bar = 125 μm. (n) Expression levels of ferroptosis related genes by qPCR in PBS‐, 20 mM‐ and 100 mM‐treated mice (*n* = 4–5); (o, p) Western blot (o) of ferroptosis related proteins in PBS‐, 20 mM‐ and 100 mM‐treated mice and the quantification (p) (*n* = 4). Data presented are means±s.e.m. *P<0.05, **P<0.01. Unpaired t test forbinary comparison. ANOVA with Fisher’s LSD for multiple comparisons.

Ferroptosis, a form of iron‐dependent regulated necrosis, has emerged as a new mode of cell death highly relevant to disease (Gao & Jiang, 2018; Stockwell et al., 2017). Ferroptosis is due to the accumulation of cellular reactive oxygen species (ROS) in excess of the redox content maintained by glutathione (GSH) and phospholipid hydroperoxidase, which uses GSH as a substrate (Xie et al., 2016). We found that Hcit causes elevated ROS levels and mitochondrial dysfunction in myocytes and further verified whether Hcit promotes ferroptosis. Ferroptosis cells have specific mitochondrial features. Figure 7a shows the ultrastructural changes in Hcit‐treated cells, with a reduction in mitochondrial volume and an increase in the density of the bilayer membrane; in some cells, the outer mitochondrial membrane was ruptured and the mitochondrial cristae disappeared, which is consistent with the ultrastructural features of the mitochondria in the muscle tissue of heat‐exposed mice. It was further found that Hcit resulted in reduced GSH levels in cells (Figure 7b). Malondialdehyde (MDA) content is an important parameter reflecting the potential antioxidant capacity of the organism and can reflect the rate and intensity of lipid peroxidation in the organism. Increasing MDA levels in myocytes with Hcit intervention (Figure 7c). Total intracellular iron and Fe^2+^ were significantly augmented in Hcit‐treated cells (Figure 7d,e). RT‐qPCR assay for ferroptosis‐related gene expression revealed that Hcit caused a decrease in myocyte *Nrf2*, *Gpx4*, *Fth1* and S*lc7a1* expression with increasing concentration (Figure 7f). In addition, Hcit intervention also resulted in reduced protein expression of *Nrf2* and *GPX4* (Figure 7g,h).

We simultaneously validated the effect of Hcit intervention on muscle tissue ferroptosis levels in mice. Consistent with the same cells, Hcit resulted in decreased GSH levels in gastrocnemius muscle (Figure 7i). Consistent with cells, Hcit resulted in a significant increase in total iron and Fe^2+^ in muscle (Figure 7j,k). We stained muscle tissue with BODIPY dye and noted an increase in stained lipid droplets in Hcit‐treated muscle (Figure 7l,m). The mRNA levels of *Nrf2*, *Gpx4*, *Fth1* and S*lc7a1* were decreased after Hcit administration (Figure 7n). Multiple protein markers were analyzed by Western blot. In consistent with control mice, mice with Hcit treatment had decreased *Nrf2* and *GPX4* levels (Figure 7o,p).

These results suggest that Hcit can lead to increased levels of ferroptosis in myocytes by inhibiting the Nrf2‐GPx4 axis, thereby promoting mitochondrial dysfunction.

### 2.7 Nrf2 activator Oltipraz inhibit Hcit‐induced ferroptosis and ameliorate saropenia

**FIGURE 8:**
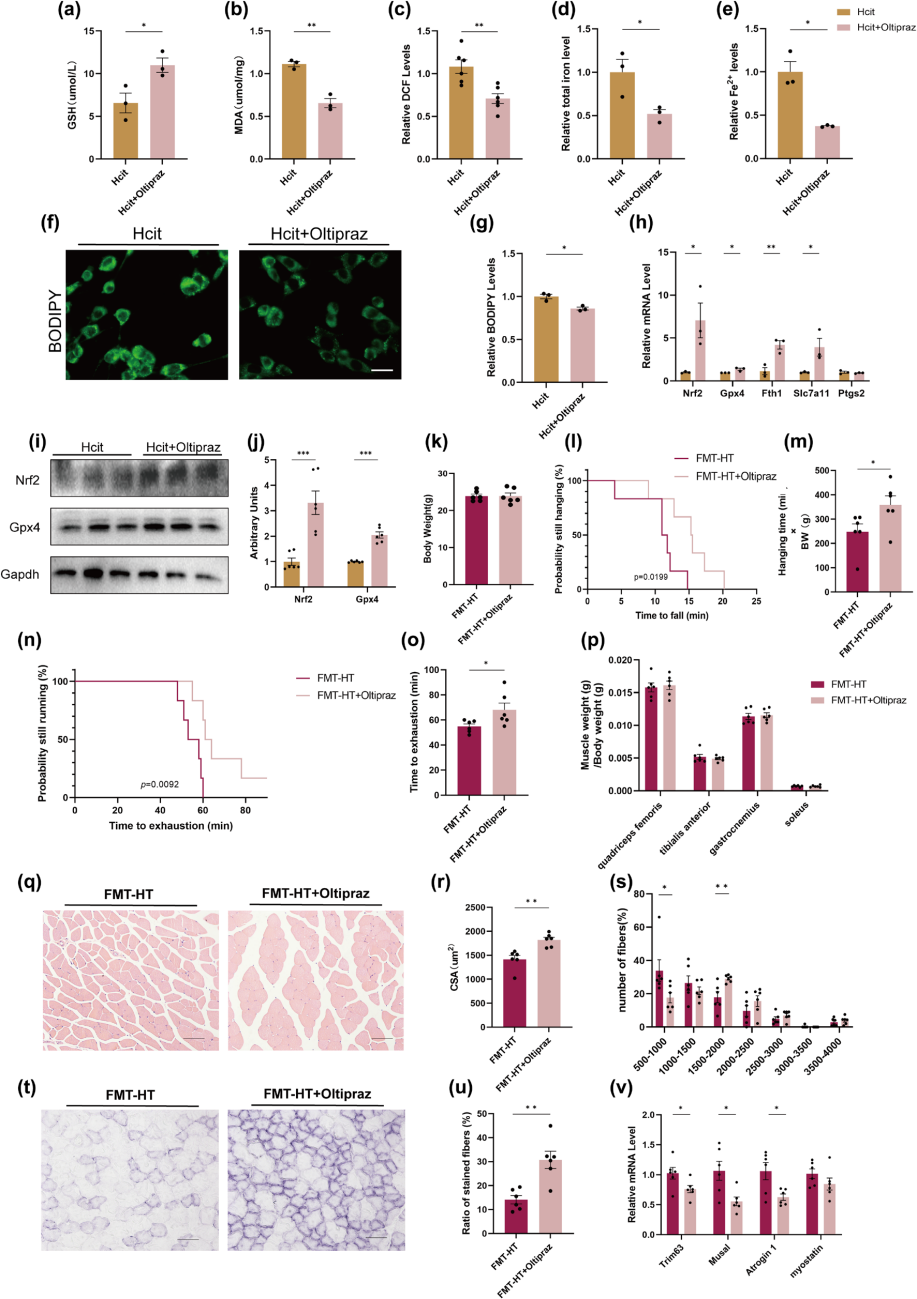
Nrf2 activator Oltipraz inhibit Hcit‐induced ferroptosis and ameliorate saropenia. (a, b) Relative GSH and MDA content in C2C12 after Oltipraz‐treated (*n* = 3); (c) Determination of ROS in Oltipraz‐treated C2C12 (*n* = 6); (d, e) Relative total iron and Fe^2+^ levels in Oltipraz‐treated C2C12 (*n* = 3); (f, g) Representative images of BODIPY staining of cross section of Oltipraz‐treated C2C12 (f) and quantification (g) of stained area (*n* = 3). Bar = 20 μm; (h) Expression levels of ferroptosis related genes by qPCR in Oltipraz‐treated C2C12 (*n* = 3); (i, j) Western blot of ferroptosis related proteins in Oltipraz‐treated C2C12 (*n* = 3); (k) Body weight of three groups mice (*n* = 6); (l, m) Determination of exercise capacity by grip test (*n* = 6); (n, o) Determination of exercise capacity by treadmill test (*n* = 6); (p) Muscle weight to body weight ratio of quadriceps femoris, tibialis anterior, gastrocnemius and soleus muscles (*n* = 6); (q‐s) Representative images of HE staining of gastrocnemius muscle cross‐section (r), quantification of mean cross‐sectional areas (s) and distribution of muscle fibers (t). Bar = 80 μm. (*n* = 6); (t,u) Representative images of succinate dehydrogenase (SDH) staining of gastrocnemius muscle cross‐section (u) and quantification (v) of stained muscle fibers. Bar = 80 μm. (*n* = 6); (v) Expression levels of muscle atrophy‐related genes by qPCR. (*n* = 6). Data presented are means±s.e.m. *P<0.05, **P<0.01, ***P<0.001. Unpaired t test forbinary comparison.

To further validate that Hcit can affect the level of ferroptosis in myocytes, we added Oltipraz, a potent activator of Nrf2, to Hcit‐intervened cells. Oltipraz resulted in increased GSH and decreased MDA, ROS, total iron and Fe^2+^ in Hcit‐intervened cells (Figure 8a–e). We stained cells with BODIPY dye, and noticed the decrease of stained lipid droplet in Oltipraz‐treated cells (Figure 8f,g). The mRNA levels of *Nrf2*, *Gpx4*, *Fth1* and S*lc7a1* were elevated after Oltipraz administration (Figure 8h). Consistently, protein levels related to ferroptosis, including *Nrf2* and *GPX4* were decreased in Oltipraz‐treated cell (Figure 8i,j).

To further investigate whether Oltipraz ameliorates sarcopenia caused by altered gut microbiota after heat exposure, we transplanted the microbiota of HT mice into GF mice by FMT experiments and administered Oltipraz and control treatments to the mice during this period. After 3 weeks, there was no significant difference in body weight between (Figure 8k) the two groups and the Oltipraz‐treated mice had increased grid suspension time and maximum run time (Figure 8l–o). Although there was no difference in muscle to weight ratios (Figure 8p), Oltipraz‐treated mice had an increased CSA of gastrocnemius muscle fibers (Figure 8q,r), a reduced proportion of small fibers (500–1000 μm^2^), and an increased proportion of medium fibers (1500–2000 μm^2^) (Figure 8s). SDH staining showed increased oxidised fibers in Hcit‐treated mice (Figure 8t,u). Further RT‐qPCR analyses of markers of muscle atrophy revealed that *Trim63*, *Musal*, *Atrogin* and *Myostain* expression was down‐regulated in Oltipraz‐treated mice muscles (Figure 8v).

## 3 DISCUSSION

In this study, we confirmed a causal relationship between heat exposure and sarcopenia in mice model. Alterations in the gut microbiota mediate this process and increase circulating levels of Hcit, leading to mitochondrial dysfunction in muscle tissue. Subsequently, Hcit was found to mediate mitochondrial dysfunction by promoting ferroptosis in C2C12 cell lines and mice. Further we verified that supplementation with Oltipraz alleviated myocyte ferroptosis induced by Hcit exposure, and ameliorate sarcopenia in FMT‐HT mice.

Increased exposure to extreme heat due to climate change (Watts et al., 2021) and urban heat island (UHI) effect (Manoli et al., 2019). Exposure to dangerously high temperatures endangers urban health and development, leading to reduced labour productivity and economic output and increased morbidity and mortality (Burke et al., 2015; Tuholske et al., 2021). Several studies have shown that acute heat exposure can affect muscle health, but the effects of chronic heat exposure on muscle function are not yet known. Therefore, we constructed models exposed to room temperature (25°C) and high temperature (37°C), respectively, and set up paired groups of hyperthermic mice on a diet. After 14 days of heat exposure, the body weights of both mice in the HT group and FR mice decreased, but the myasthenic index of the FR mice did not increase significantly, thus ruling out the effect of heat exposure on muscle through altered feeding.

There is growing evidence that the gut microbiota is involved in regulating muscle physiology in various muscular dystrophy diseases. Sarcopenia has a high prevalence in the elderly and may be affected by dysbiosis of the gut microbiota (Bindels et al., 2012; Siddharth et al., 2017). We found consistent results with previous studies by taking faecal 16 s rDNA sequencing that the dominance of *Muribaculaceae* in the microbiota of heat‐exposed mice was suppressed. Further, serum metabolomics by LC–MS revealed a correlation between Hcit and gut microbiology in heat‐exposed mice. Hcit levels were elevated in the serum of patients with severe myasthenia gravis. Our study suggests that Hcit mediates muscle mitochondrial dysfunction and exacerbates muscle atrophy in heat‐exposed mice. To investigate the importance of microbiota changes during heat exposure, we constructed FMT‐HT and FMT‐RT mice and found that the gut microbiota of heat‐exposed mice induced sarcopenia.

Ferroptosis is a recently discovered form of cell death characterised by iron‐dependent cell death associated with a demand for redox‐active iron (Dixon et al., 2012). A growing body of research suggests that ferroptosis contributes to the physiological and pathological processes of a wide range of diseases including cancer (Yang et al., 2014), neurodegenerative diseases (Skouta et al., 2014), hepatic and cardiac ischaemia/reperfusion injury (Yang & Stockwell, 2016) and acute renal failure (Linkermann et al., 2014; Proceedings of the National Academy of Sciences of the United States of America, 1990). In addition, iron overload has been found to induce ferroptosis in haemochromatosis and cardiomyopathy (Wang et al., 2017). Increasing evidence supports the role of ROS accumulation and reduced endogenous antioxidant mechanisms in the progression of sarcopenia (Kozakowska et al., 2015; Scicchitano et al., 2018). Previous studies have reported an association between muscle iron accumulation, ROS production and muscle wasting (DeRuisseau et al., 2013; Kobak et al., 2018). In our study, we firstly observed the ultrastructural changes of gastrocnemius muscle of mice in the HT group and Hcit‐treated cells by electron microscope scanning, and found that the mitochondrias decreased in volume and the density of the bilayer membrane increased; some mitochondrias showed a reduction and disappearance of cristae, and a fragmentation of the outer membrane, which was consistent with the morphology of ferroptosis mitochondria. Then we found that the increase in Hcit can mediate the mitochondrial ferroptosis phenotype in myocytes. Hcit leads to elevated levels of cellular lipid peroxidation and exacerbates mitochondrial dysfunction by inhibiting the Nrf2‐GPx4 axis.

Efforts have been made to address diseases associated with ferroptosis. Studies have shown that overexpression of Nrf2 by the Nrf2 activator Oltipraz significantly upregulates antioxidant response elements (AREs) and inhibits iron deposition in osteoblasts. Furthermore, the combined use of ferroprostin‐1 and Oltipraz significantly improved the severity of iron deposition and osteolysis in an animal model of particles induced osteolysis (Xu et al., 2021). Oltipraz also attenuates cerebral ischaemia‐reperfusion injury by inhibiting oxidative stress and iron death in mice (Jian et al., 2024). However, studies on the alleviation of sarcopenia by Oltipraz through the inhibition of ferroptosis are not yet known. In our results, the use of Oltipraz reversed the ferroptosis effect of Hcit on cells. Oltipraz treatment of FMT‐HT mice was further shown to ameliorate the effects of heat exposure on muscle function in mice.

In conclusion, our findings reveal a facilitating effect of heat exposure on sarcopenia and provide new perspectives on the gut‐muscle axis.

## 4 MATERIALS AND METHODS

### 4.1 Animals

All the mice used in our experiments were male. Wild‐type C57BL/6J mice were purchased from Shanghai SLAC Laboratory Animal Co Ltd. (Shanghai, China). All mice were kept on a C57BL/6J background and maintained in a standard, pathogen‐free facility at the Laboratory Animal Research Centre of Central South University, with temperatures of 22–24°C in all groups except the high‐temperature‐exposure model group, with a 12‐h dark/light cycle, and the health status of all mice was monitored daily. In this study, all 12‐month‐old size male mice were used. All mice were fed a standard normal food diet purchased from Shanghai Laboratory Animal Co Ltd. (Shanghai, China), and room temperature control mice and heat‐exposed mice had ad libitum access to standard food and water. All animal care protocols and experiments were reviewed and approved by the Animal Care and Use Committee of the Laboratory Animal Research Centre, Xiangya School of Medicine, Central South University, and complied with all relevant ethical norms for animal research.

### 4.2 Temperature treatment

12‐month‐old mice were randomly divided into three groups and intervened in the corresponding temperature environments for 14 days. RT group mice were exposed to room temperature (22°C). HT group mice were exposed to a 37°C hyperthermia chamber and had normal access to food or water at will. Mice in the FR group were exposed to room temperature and kept their food intake the same as that of the HT group.

### 4.3 Metabolic parameter measurements

Metabolic parameter experiments were conducted with a Comprehensive Lab Animal Monitoring System (CLAMS, Columbus Instruments). Mice in the CLAMS were maintained at room temperature (25°C) and experiments continuously for 48 h. Results of oxygen consumption, energy expenditure, and food intake were collected and analyzed.

### 4.4 Feces microbiota transplantation (FMT)

Before transplantation, mice were given an antibiotic cocktail containing ampicillin 1 gL‐1, neomycin 0.5 gL‐1, vancomycin 0.5 gL‐1, and metronidazole 1 gL‐1 for 1 week in 200 μL (by gavage after 6 h of fasting each day) thereby moving the microbiota in the gut. 5–6 fresh feces pellets from RT and HT mice were collected for the microbiota suspension preparation. The faeces were resuspended with 1 mL PBS (125 mg:1 mL). The suspension was organized as the transplantation vehicle by vortex vigorously for 1 min and centrifuging at 800 g for 8 min. Receptor mice (FMT‐RT and FMT‐HT) were given 150 μL of the fecal microbiota solution by gavage three times a week for 3 weeks. Oltipraz (30 mg/kg) dissolved in 40% polyethyleneglycol 400 was orally administered to mice three times per week during the feces microbiota transplantation. Control animals received vehicle only.

### 4.5 Hcit treatment

12‐month‐old mice were randomly divided into three groups. Mice were injected intraperitoneally with 100μL of Hcit at concentrations of 20 mM and 100 mM thrice a week for 3 weeks. The control mice were injected with an equal amount of PBS.

### 4.6 Skeletal muscle function tests

#### 4.6.1 Grid test

Mice were placed in the center of the wire grid system, and the grid was rotated to an inverted position over 2 s, with the mouse head declining first. The grid was held steadily 40–50 cm above a padded surface. Muscle strength is determined by the amount of time spent hanging on the grid and the amount of time (min) × body weight (g).

#### 4.6.2 Treadmill

Mice first ran at 10 m/min for 5 min in consecutive 3 days. For the treadmill test, the speed was increased by 2 m/min every 2 min until 24 m/s. The criterion of exhaustion was defined as the inability of the animal to run on the treadmill for 5 s despite mechanical prodding.

### 4.7 Measurement of ROS level, ATP content, mitochondrial membrane potential (MMP)

ROS was measured using the fluorescent probe DCFH‐DA (Beyotime, S0033S). In vivo, 20 mg of gastrocnemius muscle was minced and then digested with type I collagenase for 60 min. The digestion was ended by adding an equal medium volume with 2% FBS, filtered through a 100 μm cell strainer, and centrifuged at 200 × g for 5 min. The precipitate was separated and loaded with DCFH‐DA. In vitro, cells were digested with trypsin, digestion was terminated by adding an equal volume of serum‐containing medium, and cells were collected at 1000 rpm and loaded with probes. Fluorescence was detected using a Microplate Reader (Envision).

ATP was determined using the Enhanced ATP Assay Kit (Beyotime, S0027). Cell levels were lysed at 100ul lysate per 12‐well plate cell, and muscle tissue was lysed using 200ul lysate per 20 mg of tissue. After lysis, centrifugation was performed at 4°C 12000 g for 5 min, and the supernatant was removed and followed as directed. The luminescence was further detected using a Microplate Reader (Envision).

Mitochondrial membrane potential (ΔψM) was detected by the JC‐1 probe (Beyotime, C2006) and TMRE probe (Beyotime, C2001S). Firstly, a mitochondrial extraction kit (Beyotime, C3606) was used to isolate the mitochondria of muscle or C2C12 cells to obtain mitochondrial resuspension. After incubating 100 μg mitochondria with a JC‐1 probe, JC‐1 fluorescence was detected by the Envision microplate reader. TMRE testing was performed with probe incubation as instructed.

### 4.8 16S rDNA sequencing

Four to five RT and HT mice feces were collected, and total genomic DNA was extracted using a DNA extraction kit. The purity and concentration of the extracted DNA were tested by agarose gel electrophoresis. The V3‐V4 region of bacterial DNA (5′‐TACGGRAGGCAGCAG ‐3′, 5′‐GGGTATCTAATCCT‐3′) was amplified with primers. PCR products were purified with AMPure XP beads (Beckman) and further adjusted for sequencing. DNA was sequenced using Illumina NovaSeq6000 from OE Biotech (Shanghai). All representative reads were annotated and analyzed against the Silva database (Version 132) using the RDP classifier (confidence threshold of 70%).

### 4.9 Non‐targeted metabolomics sequencing

Plasma samples were collected and immediately stored at−80°C. Samples were thawed on ice and processed to remove proteins prior to LC–MS/MS analysis. Samples were detected using a liquid‐mass spectrometry system consisting of a Waters ACQUITY UPLC I‐Class plus/Thermo QE ultra‐performance liquid tandem high‐resolution mass spectrometer.

### 4.10 Transmission electron microscopy

Transmission electron microscopy (TEM) analyzed the gastrocnemius mitochondria and C2C12 cell line morphology. Gastrocnemius muscle was cut into 1.0 mm3 blocks and fixed overnight in 2.5% glutaraldehyde (servicebio). After rinsing thrice, the designated tissue blocks were incubated with 1% osmium tetroxide. The tissue was then dehydrated and embedded in epoxy resin using a series of graded ethanol. The embedded tissues were cut into ultrathin sections (60–80 nm) before image acquisition, stained with 2% uranyl acetate and lead citrate, and observed by transmission electron microscopy. The cultured C2C12 cells were digested with trypsin. After termination of digestion, the above cell suspension was collected in 1.5 mL EP tubes; the cell precipitate was collected by centrifugation at 1000 rpm for 5 min at 4°C. The supernatant of the medium was carefully aspirated and discarded. The electron microscope fixative 1 mL was added gently along the wall without blowing away the cell clusters, and the cells were left to send the samples at 4°C.

### 4.11 Cell

C2C12 myoblasts (CL‐0044, Procell) were cultured in Dulbecco's modified Eagle's medium (DMEM, Gibco) including glucose 25 mM, 10% fetal bovine serum (FBS, Gibico), 100 U/mL of penicillin and 100 μg/mL of streptomycin (Procell). All cells were cultured at 37°C under a 5% CO_2_ humidified atmosphere. Cells were treated with Hcit (0.5 mM, 1 mM or 2 mM), or Oltipraz (50 μM) for 24 h.

### 4.12 Serum epinephrine, cortisol and vasopressin measurements

Serum epinephrine, cortisol and vasopressin were measured by epinephrine ELISA kit (CUSABIO, CSB‐E08679m), cortisol ELISA kit (CUSABIO, CSB‐E05113m), and vasopressin ELISA kit (CUSABIO, CSB‐E09272m) respectively.

### 4.13 GSH levels and MDA levels

Glutathione levels were measured using the Reduced Glutathione (GSH) Colourimetric Assay Kit (Elabscience, E‐BC‐K030‐M). For muscle tissue, 30 mg of gastrocnemius was taken and added to 270 ul of PBS to take the supernatant. 0.1 mL of reagent was added and mixed well, then centrifuged at 4500 × g for 10 min. 0.1 mL of supernatant was taken, and the test was performed according to the operation table in the instruction manual. For cells, 10^6^ cells were taken and homogenised by adding 300–500 μL of PBS (0.01 M, pH 7.4). After homogenisation, the cells were centrifuged at 10,000*g* for 10 min at 4°C, and the supernatant was placed on ice. The supernatant was placed on ice. The fluorescence was detected using a Microplate Reader (Envision).

MDA level was measured using a Malondialdehyde (MDA) assay kit (Beyotime, S0131S). For cells, 0.1 mL of lysate is used per 10^6^ cells. The supernatant was obtained by centrifugation of homogenate or pyrolysis at 12,000 g for 10 min for subsequent determination. The fluorescence was detected using a Microplate Reader (Envision).

### 4.14 Total iron and Fe^2+^ measurements

Cell ferrous iron and muscle ferrous iron were measured by Cell Ferrous Iron Colorimetric Assay Kit (Elabscience, E‐BC‐K881‐M) and Ferrous Iron Colorimetric Assay Kit (Elabscience, E‐BC‐K773‐M) respectively. Cells and muscles total iron were measured by Cell Total Iron Colorimetric Assay Kit (Elabscience, E‐BC‐K880‐M) and Total Iron Colorimetric Assay Kit (Elabscience, E‐BC‐K772‐M) respectively, according to the manufacturer's instructions.

### 4.15 RNA extraction, reverse transcription, and real‐time qPCR

Cellular and muscle tissue total RNA were prepared using the TRIzol method. The cDNA preparation kit (Takara) using reverse transcription technique was used for RT‐qPCR by ABI QuantStudio3 according to the manufacturer's instructions. The primer pairs used for RT‐qPCR are listed in Table 1.

**TABLE 1:** The primer pairs used for RT‐qPCR.

Target gene	Sense primer	AntisensePrimer
Trim63	5′‐CATCTTCCAGGCTGCGAATC‐3′	5′‐ACTGGAGCACTCCTGCTTGT‐3′
Musal	5′‐CTTCAGTCTCGTGGAATGGTAATCTT‐3′	5′‐TGCAGTACTGAATCGCCATAC‐3′
Atrogin1	5′‐CAGAGAGGCAGATTCGCAAG‐3′	5′‐GGTGACCCCATACTGCTCTC‐3′
myostatin	5′‐AGTGGATCTAAATGAGGGCAGT‐3′	5′‐GTTTCCAGGCGCAGCTTAC‐3′
Nrf1	5′‐AGCACGGAGTGACCCAAAC‐3′	5′‐TGTACGTGGCTACATGGACCT‐3′
Tfam	5′‐ATTCCGAAGTGTTTTTCCAGCA‐3′	5′‐TCTGAAAGTTTTGCATCTGGGT‐3′
Ppargc‐1α	5′‐CCCTGCCATTGTTAAGACC‐3′	5′‐TGCTGCTGTTCCTGTTTTC‐3′
Drp1	5′‐TTACGGTTCCCTAAACTTCACG‐3′	5′‐GTCACGGGCAACCTTTTACGA‐3′
Fis1	5′‐TGTCCAAGAGCACGCAATTTG‐3′	5′‐CCTCGCACATACTTTAGAGCCTT‐3′
Mfn1	5′‐CCTACTGCTCCTTCTAACCCA‐3′	5′‐AGGGACGCCAATCCTGTGA‐3′
Opa1	5′‐TGGAAAATGGTTCGAGAGTCAG‐3′	5′‐CATTCCGTCTCTAGGTTAAAGCG‐3′
Becn1	5′‐CCGCGGTAGAACGAGCC‐3′	5′‐AAGTAATGGAGCTGTGAGTTCCT‐3′
Atg5	5′‐GGAGAGAAGAGGAGCCAGGT‐3′	5′‐GCTGGGGGACAATGCTAATA‐3′
Pik3c3	5′‐GTGAAGTACCCTGACCTGCC‐3′	5′‐AGTCATGCATTCCTTGGCGA‐3′
Park2	5′‐CCGAATCACCTGACGGTTCA‐3′	5′‐TCTGGCTGCTTCTGAATCCC‐3′
Sod‐1	5′‐TGGTGGTCCATGAGAAACAA‐3′	5′‐AATCCCAATCACTCCACAGG‐3′
Gpx1	5′‐AGTCCACCGTGTATGCCTTCT‐3′	5′‐GAGACGCGACATTCTCAATGA‐3′
Cat	5′‐GGAGGCGGGAACCCAATAG‐3′	5′‐GTGTGCCATCTCGTCAGTGAA‐3′
TNFa	5′‐CATCTTCTCAAAATTCGAGTGACAA‐3′	5′‐TGGGAGTAGACAAGGTACAACCC‐3′
IL1β	5′‐ATGATGGCTTATTACAGTGGCAA‐3′	5′‐GTCGGAGATTCGTAGCTGGA‐3′
Nrf2	5′‐CCAGAAGCCACACTGACAGA‐3′	5′‐CCGTCCAGGAGTTCAGAGAG‐3′
Gpx4	5′‐AGGCAGGAGCCAGGAAG‐3′	5′‐CCTTGGGCTGGACTTTC‐3′
Fth1	5′‐GAGCCCTTTGCAACTTCGTC‐3′	5′‐CCGGTCAAAATAACAAGACATGG‐3′
Slc7a11	5′‐GGCACCGTCATCGGATCAG‐3′	5′‐CTCCACAGGCAGACCAGAAAA‐3′
Ptgs2	5′‐CCGGTCAAAATAACAAGACATGG‐3′	5′‐TCCAGGAGGATGGAGTTGTT‐3′

### 4.16 Western blotting

Muscle tissue and cells were lysed in RIPA Lysis Buffer (Biosharp), loaded on an 8%–15% SDS‐PAGE gel, and transferred to the PVDF membrane (Millipore). Membranes were blotted with targeted primary antibodies as follows: anti‐NRF2 (proteintech,16,396‐1‐AP, 1:2000), anti‐GPX4 (proteintech,67,763‐1‐Ig,1:1000), anti‐GAPDH (OriGene, TA802519, 1:2000). The blots were incubated again the next day with the corresponding secondary antibodies (Invitrogen, 31,430 and 31,460, 1:2000), visualised using Western ECL substrate (BioRad) and quantified using Image Lab software (BioRad).

### 4.17 H&E, SDH, TMRE and BODIPY staining

For Hematoxylin and eosin (H&E) staining, gastrocnemius tissues were soaked in 4% formaldehyde for 24 h, then paraffin embedding, slicing, hematoxylin and eosin staining (Servicebio).

For SDH staining, the isolated fresh gastrocnemius were embedded in OCT and then quickly frozen, and then sections with a thickness of 8 μM were prepared and stained with SDH solution (Solarbio).

For TMRE staining, 24‐well C2C12 cell slivers were prepared first After the cells grew to the appropriate density and were treated with drug intervention for 24 h. The dye solution was prepared using the ratio of 1mlTMRE diluent and 1ulTMR probe, and then each cell well was incubated with 500μL system at 37°C for 30 min. After incubation, the tablets were washed with PBS for 3 times and sealed.

For BODIPY staining, gastrocnemius tissues frozen slices or C2C12 cell slivers were staining with BODIPY dye (3.8 μM working solution, Invitrogen).

Figures were analysed by Image J processing software.

### 4.18 Statistical analysis

All data are expressed as means ± SEM. The statistical significance of the differences between various treatments or groups was measured by either Student's *t*‐test or analysis of variance.

(ANOVA) followed by the Bonferroni post‐test. Data analyses were performed using GraphPad Prism 7.0. *p* values assumed two‐tailed distribution and unequal variances (*p < 0.05; **p < 0.01; ***p < 0.001).

## AUTHOR CONTRIBUTIONS

Zhu‐Ying Xia and Ping Yin designed this study; Yi‐Fan Guo and Zhe‐Yu Liu performed most of the experiments, generated data, analyzed the data and designed the figures, and wrote the manuscript; Min Zhou, Wei‐Hong Kuang, Ya Liu, Yan Huang, Ping Yin and Zhu‐Ying Xia supervised this study and revised the manuscript.

## FUNDINGINFORMATION

This work was supported by grants from the National Natural Science Foundation of China (NO. 82370883).

## CONFLICT OF INTEREST STATEMENT

The authors declare that they have no conflict of interest.

## Supporting information


**Figure S1.** (a) Oxygen consumption of mice. *n* = 4 biologically independent animals per group. (b) Average energy expenditure. *n* = 4 biologically independent animals per group. (c) Daily food intake of RT, FR and HT mice. *n* = 4 biologically independent animals per group. (d–f) Determination of epinephrine, cortisol and vasopressin in serum by ELISA. *n* = 5 biologically independent animals per group. (g) Expression levels of muscle atrophy‐related genes by qPCR in 0, 10 , 20 and 100 μM Difelikefalin‐treated C2C12. (*n* = 3).

Table S1.

## Data Availability

The data that support the findings of this study are available from the corresponding author upon reasonable request.
