# Supplementary figures and images for: Heat exposure promotes sarcopenia via gut microbiota‐derived metabolites
Source: Aging Cell. 2024 Oct 29;24(2):e14370. doi: 10.1111/acel.14370 (PMC11822625; doi:10.1111/acel.14370)

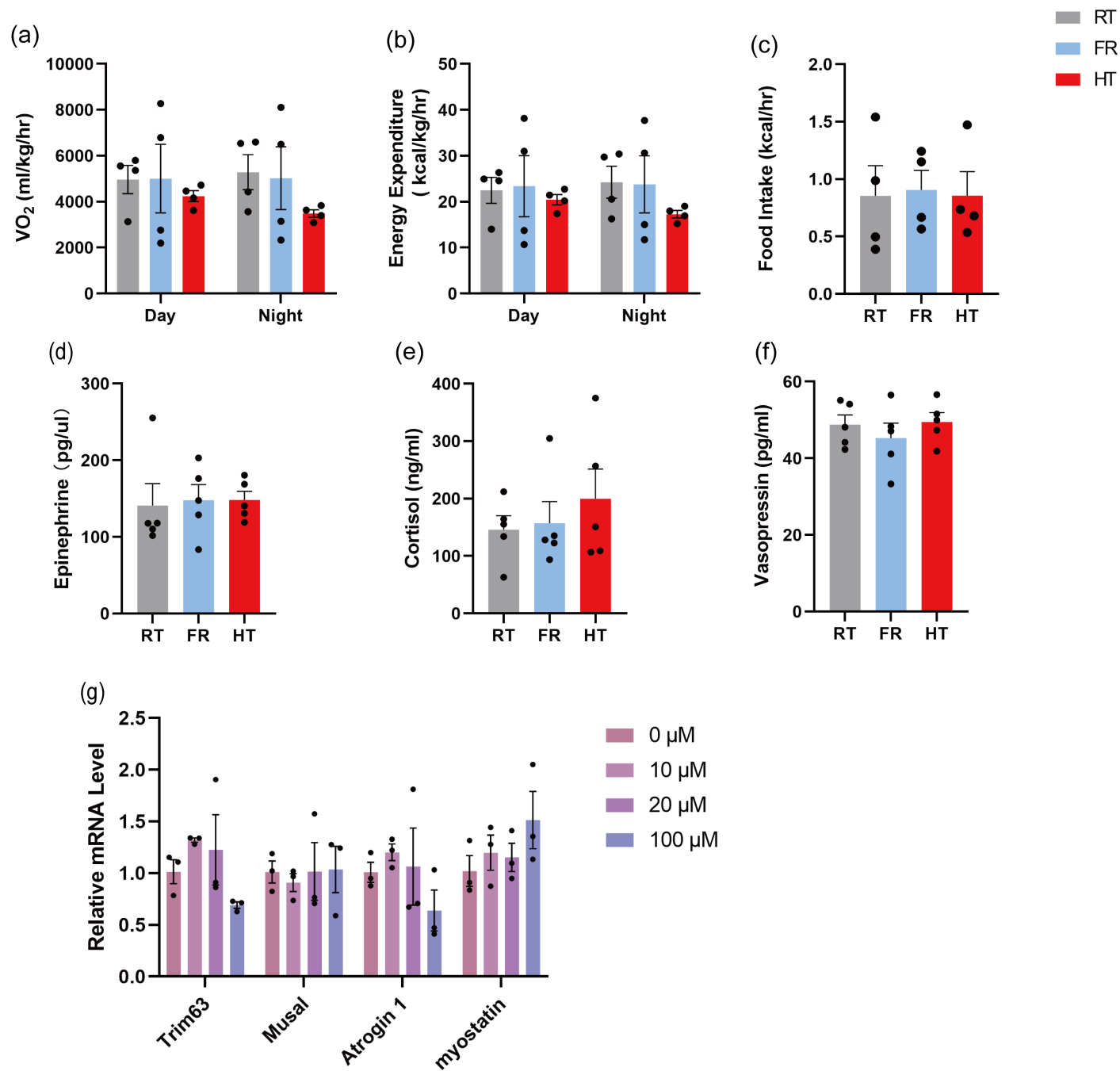

Supplement: Supplementary file 1 — Figure S1. (a) Oxygen consumption of mice. n = 4 biologically independent animals per group. (b) Average energy expenditure. n = 4 biologically independent animals per group. (c) Daily food intake of RT, FR and HT mice. n = 4 biologically independent animals per group. (d–f) Determination of epinephrine, cortisol and vasopressin in serum by ELISA. n = 5 biologically independent animals per group. (g) Expression levels of muscle atrophy‐related genes by qPCR in 0, 10 , 20 and 100 μM Difelikefalin‐treated C2C12. (n = 3). [file ACEL-24-e14370-s001.pdf]
